# Is exercise addiction in fitness centers a socially accepted behavior?

**DOI:** 10.1016/j.abrep.2017.09.002

**Published:** 2017-09-27

**Authors:** Mia Beck Lichtenstein, Bolette Emborg, Simone Daugaard Hemmingsen, Nina Beck Hansen

**Affiliations:** a Department of Psychology, University of Southern Denmark, Centre for Telepsychiatry, J. B. Winsløws Vej 18, 220 B, DK-5000 Odense C, Denmark; b Aarhus University, Bartholins Allé 2, DK-8000 Aarhus C, Denmark; c Odense University Hospital, Mental Health Services in the Region of Southern Denmark, Sdr. Boulevard 29, DK-5000 Odense C, Denmark; d Department of Psychology, University of Southern Denmark, Campusvej 55, DK-5230 Odense M, Denmark

## Abstract

**Background:**

Fitness exercise is popular and associated with improved health and social status. Taken to extremes, however, exercise can become an addiction. One suggested symptom of exercise addiction is “conflicts” with family and friends. However, it may be difficult to recognize excessive exercise patterns if they are accepted and encouraged by relatives. The aim of this study was to explore if fitness exercisers with a high risk of addiction experienced the same level of exercise support as exercisers with a low risk of addiction. Furthermore, we wanted to examine if social support affected the subjective reporting of “conflicts”.

**Method:**

A total of 577 fitness exercisers completed the Exercise Addiction Inventory (EAI) and two questions asking about “exercise support from family and friends” and “present eating disorder”.

**Results:**

Exercisers at high risk of exercise addiction reported the same level of support from relatives as those at low risk. Exercisers with high levels of exercise support reported significantly fewer conflicts, even if they were at high risk of addiction. If an eating disorder was present, the level of exercise support was significantly reduced.

**Conclusion:**

Exercise addiction might be difficult to identify with the general behavioral addiction symptom “conflict”, since exercise is socially accepted even in subjects with high risk of exercise addiction. If an eating disorder is present, the exercise routines seem to be interpreted as socially undesirable. Screening for exercise addiction with the EAI should take into account that fitness exercisers rarely report conflicts, which could result in false negative cases.

## Highlights

• Exercise addiction seems to be a socially accepted behavior.
• Exercise support might affect reliability of addiction measures.
• Exercise behavior in eating disorders receive less social acceptance.

## 1 Introduction

The number of adults who perform regular exercise has increased significantly during the last few decades (Overgaard et al., 2014). In particular, exercise in fitness centers is popular (Fester and Gottlieb, 2017, Kirkegaard, 2007). A simple Google search on ‘fitness’ plus ‘exercise’ gives 281.000.000 results of fitness trends, fitness programs, workout videos and guides. Fitness centers aim to engage people in regular exercise, and instructors are educated in motivating fitness members to push themselves to their physical limits.

However, there may be a downside to striving for physical fitness and bodily recognition. Addiction to exercise has been described as excessive and obsessive exercise patterns that may result in physical injury and overload (Hausenblas & Downs, 2002). Prevalence proportions of exercise addiction in fitness center attendees have been found to range from 2% in Hungary (Menczel et al., 2013), 5–6% in Denmark (Lichtenstein et al., 2014, Lichtenstein and Jensen, 2016) and 30% to 42% in France (Lejoyeux et al., 2008, Lejoyeux et al., 2012). The different prevalences reported probably reflect differences in samples and operationalizations of fitness exercise addiction.

Based on Brown's theory of behavioral addictions (Brown, 1997), the following six components have been suggested to define exercise addiction (Griffiths, 1997): *Salience* (exercise is the most important thing in life), *conflicts* (interpersonal conflicts about the harm the excessive activity may be doing and/or intrapsychic conflicts), *mood modification* (a coping strategy to regulate emotions), *tolerance* (an increasing amount of exercise is needed to achieve a psychological effect), *withdrawal symptoms* (e.g. irritability when exercise is reduced or when missing a workout) and *relapse* (reversion to earlier exercise patterns in spite of attempts to reduce exercise).

The Exercise Addiction Inventory (EAI; Terry, Szabo, & Griffiths, 2004) was developed to include these six components. The EAI-item measuring conflict was phrased to only include interpersonal problems: “Conflicts have arisen between me and my family and/or my partner about the amount of exercise I do”. All items are rated on a 5-point Likert scale ranging from *strongly disagree* (1) to *strongly agree* (5). The cut-off score indicating a high risk of addiction is ≤ 24. The EAI has shown good psychometric properties in Danish fitness exercisers (Lichtenstein et al., 2014, Lichtenstein et al., 2014) and across countries (Griffiths et al., 2015). Testing the internal structure of the scale, results reveal a one-factor solution in these studies. However, the EAI item measuring “*conflicts with family and partner about exercise*” was found to have poorer psychometric value compared to the other items in three Danish EAI studies (Lichtenstein et al., 2014, Lichtenstein and Jensen, 2016, Lichtenstein et al., 2014), even though the differences in factor loadings were very small.

In comparison with other addictive conducts (e.g. alcohol, gambling, or binge eating), exercise is a socially accepted behavior, *possibly* even when taken to extremes. The striving for a lean and fit body is usually perceived as a sign of a healthy lifestyle and personal success, and family and friends may accept and encourage fitness exercisers to maintain excessive exercise habits. Thus no conflicts arise, which may affect the predictive value of the EAI item measuring “conflicts”.

The distinction between a healthy commitment to fitness versus a harmful exercise addiction is difficult to conceptualize if current screening instruments (e.g. EAI) are not sensitive to the specific nature of the phenomenon. Yet no studies have explored whether self-reported exercise support from relatives and friends affects the responses to the EAI-conflict item.

Similarities have been found between exercise addiction and eating disorders in terms of obsessive exercise patterns, controlling body shape, and perfectionist personality traits (Bamber et al., 2000, Bamber et al., 2003, Lichtenstein et al., 2014, Veale, 1987). Muscle dysmorphia is a variant of body dysmorphic disorder characterized by beliefs of insufficient muscularity, and engagement in excessive muscle-building activities such as weightlifting and using anabolic steroids (Tod, Edwards, & Cranswick, 2016). Athletes with muscle dysmorphia often have low body fat and disordered eating habits. The syndrome can be thought to have commonalities with compulsive exercise due to the extreme focus on achieving fitness goals, but the relationship has not yet been investigated.

Eating disorders and body dysmorphic disorder are recognized as psychiatric disorders in the diagnostic manuals (American Psychiatric Association, 2013, World Health Organization, 1992), whereas exercise addiction is not. However, the two conditions often appear simultaneously, and Davis and Claridge proposed that eating disorders should be understood as addictions or that addictions have high comorbidity with eating disorders (Davis & Claridge, 1998). They found that addictive and obsessive-compulsive traits were associated with weight preoccupation and excessive exercise in patients with eating disorders.

It could be hypothesized that excessive exercise patterns connected with disturbed eating are perceived more undesirable and thus lead to less exercise support from family and friends.

This study contributes with a preliminary examination of possible differences in perceived exercise support between a group of fitness exercisers with a high risk of exercise addiction (EAI score ≤ 24) and low risk of exercise addiction (EAI score > 24). We explored whether perceived exercise support affected the ratings on the EAI-conflict item, and if the co-occurrence of an eating disorder affected exercise support.

## 2 Material and methods

Data for this cross-sectional study were collected through a questionnaire survey among fitness members at two large fitness centers in Denmark. Paper questionnaires were available in the reception areas of the centers, and an online version of the survey was accessible on each center's Facebook Group. All responses were anonymous as no personal identification information was requested. A total of 577 fitness exercisers completed the survey. The gender distribution was 71% females, and the mean age was 26.7 years (range 15–73 years).

We used the Danish version of the EAI to identify participants at risk of exercise addiction.

An EAI score of 24–30 points indicates a high risk of exercise addiction, while scores of 6–23 indicate low risk. The conflict-item was phrased as: “*Conflicts have arisen between me and those around me (e.g. family, partner, friends, colleagues) about the amount of exercise I do*”.

In addition, we collected information on age, gender and weekly exercise amounts rated in categories of: 0–3, 4–7, 8–11, 12–15, and ≥ 16 hours per week.

Participants were asked to rate their perceived feeling of social support in their current exercise routines, phrased as: "Those around me (e.g. family, partner, friends, colleagues) supports me 100% in my exercise routines", and to rate their perceived addiction to exercise, phrased as: "I see myself as addicted to exercise". These statements were rated on a 5-point Likert scale from totally disagree (1) to totally agree (5). We further asked the participants to indicate if they continued to exercise despite injury or illness (yes/no), and if he/she suffered from an eating disorder, e.g. anorexia nervosa or bulimia nervosa (yes/no).

### 2.1 Statistical analyses

Data were analyzed using the Statistical Package for the Social Sciences (SPSS) version 24. To examine potential differences between the groups of fitness members with high versus low risk of exercise addiction, we used *t*-test for continuous data (amount of weekly exercise, perceived exercise addiction, and perceived exercise support) and a chi-square test for dichotomous data (exercising despite injury/illness and self-reported eating disorder).

To explore an association between “exercise support” and “conflicts”, two groups were created based on the scores on the exercise support item: one group with high levels of exercise support (Likert ratings 4–5) and one group with low levels of exercise support (Likert ratings 1–3). The two groups were compared in their ratings on the EAI-conflict item, risk of exercise addiction, and self-reported eating disorder. A *t*-test was used for continuous data (conflict-item) and a chi-square test for dichotomous data (risk of exercise addiction and self-reported eating disorder).

## 3 Results

### 3.1 High or low risk of exercise addiction

Descriptive characteristics of the two addiction groups are presented in Table 1. In total, 6.8% (n = 39) of the participants were categorized as being at high risk of exercise addiction (total EAI score 24–30).

**Table 1:** Characteristics of fitness exercisers with a high versus low risk of exercise addiction.

Dichotomous data	High risk of exercise addiction n = 39	Low risk of exercise addiction n = 538	P
	% (numbers counted)	% (numbers counted)	
Gender female	71.4% (25 out of 35)	71.4% (n = 350 out of 490)	= 1.00
Exercise hours/week			> 0.001
0–3 h	0.0% (0^a^ out of 31)	16.9% (75 out of 445)	
4–7 h	19.4% (6 out of 31)	47.9% (213 out of 445)	
8–11 h	41.9% (13 out of 31)	26.3% (117 out of 445)	
12–15 h	35.5% (11 out of 31)	7.4% (33 out of 445)	
≥ 16 h	3.2% (1^a^ out of 31)	1.6% (7 out of 445)	
Exercise despite injury	61.3% (19 out of 33)	17.5% (78 out of 446)	> 0.001
Eating disorder	16.1% (5 out of 31)	2.4% (11 out of 449)	> 0.001


Continuous data	Mean (SD)	Mean (SD)	
Age	24.4 (SD = 7.4)	26.7 (SD = 8.2)	= 0.092
Perceived addiction (Likert 1–5)	3.77 (SD = 1.0)	2.46 (SD = 1.2)	> 0.001
Exercise support (Likert 1–5)	3.8 (SD = 0.9)	3.8 (SD = 0.9)	= 0.882

a Fishers Exact was used when number in cell was below 5.

As shown in Table 1, high risk of exercise addiction was associated with more weekly exercise, as 80.6% of those with high risk of addiction exercised 8 h/week or more compared to 35.3% of those with low risk of addiction. A higher proportion of those with high risk of addiction reported exercise despite injury/illness (61%), reported having an eating disorder, and had higher scores on self-perceived exercise addiction. However, our results showed no difference between the addiction groups regarding perceived exercise support from family and friends. Even when we removed the 16 cases with self-reported eating disorder, we found that the group with high risk of exercise addiction reported the same level of exercise support as the group with low risk of addiction.

### 3.2 Self-perceived exercise support and rating at the EAI items

In the total sample, 60% (n = 323) reported high levels of exercise support from family and friends compared to 40% (n = 215) reporting low levels of exercise support. No significant differences were found in gender and age between the two groups. Furthermore, we did not find any difference in the prevalence of exercise addiction in the two groups (6% versus 7% reporting exercise addiction). However, when we compared the prevalence of self-reported eating disorder, we found that only 1.6% (5 out of 317 cases) reported exercise support compared to 6.7% (11 out of 163) reporting no exercise support, p = 0.004. In the further analyses, we removed the 16 cases with self-reported eating disorder because we wanted to exclude cases with secondary exercise addiction.

The ratings for the six EAI items for the two groups are presented in Table 2. Our results indicated that the group with a high level of exercise support reported significantly fewer conflicts, whereas the rest of the EAI items were not associated with the level of social support.

**Table 2:** Fitness members with high versus low levels of perceived exercise support.

EAI items (Likert 1–5)	High levels of exercise support n = 323		Low levels of exercise support n = 215		Cohen's d
	Mean score	SD	Mean score	SD	
1 Salience	2.4	0.97	2.5	1.00	0.10
2 Conflict⁎⁎⁎	1.8	0.96	2.1	1.16	0.28
3 Mood modification	3.8	1.00	3.7	0.95	0.10
4 Tolerance	3.0	1.08	2.8	1.06	0.18
5 Withdrawal symptoms	3.3	1.14	3.3	1.18	0.16
6 Relapse	2.5	1.16	2.5	1.04	0.10
EAI total score	16.7	4.29	17.0	4.44	0.06

(Note: 16 cases with self-reported eating disorder were excluded from this analysis).

⁎⁎⁎ p < 0.001.

A brief psychometric evaluation of the EAI items showed a high reliability of the scale, as the Cronbach’s alpha coefficient was 0.78.

## 4 Discussion

In the area of addiction disorders, conflicts or interpersonal problems with friends, families or at work about the amount of substance use (e.g. alcohol) or the type of activity (e.g. gambling) is considered a diagnostic sign of addiction (American Psychiatric Association, 2013) In Brown's theory of behavioral addictions (Brown, 1997), “conflict” is described as one of the most important classical psychological features of behavioral addiction: “The addictive activity produces ill-feeling and disputes with people immediately around the addict about the harm the excessive activity may be doing to the addict and to all concerned”.

We questioned whether the EAI item assessing “conflicts with families and friends” was reliable in measuring exercise addiction, as exercise is considered a healthy activity in contrast to drinking and gambling.

The results of the current study indicate that exercise addiction may be a socially accepted behavior, as we did not find a significant difference in the level of perceived exercise support from family and friends between those with a high risk of exercise addiction and those with a low risk of addiction. This suggests that addictive exercise behavior is considered to lie within cultural norms and is therefore socially supported and encouraged. Some authors have also distinguished between unproductive and productive behavioral addictions (Andreassen et al., 2013). Unproductive behavioral addictions include gambling, shopping, and social media addictions, whereas productive behavioral addictions include exercise addiction. The word productive indicates that it is more socially accepted, i.e. society views it as productive. However, the behavior has become maladaptive and has great implications for the affected individual.

We found that fitness exercisers who felt supported by their family and friends had significantly lower scores on the EAI conflict-item. The reliability of the conflict-item can thus be questioned, as the risk of false negative cases increases when the conflict-item is rated lower than the other items, and thus decreases the total EAI score. However, a comparison between this specific conflict item and self-reported social support may be redundant. People with less perceived social support from family and friends are likely to score higher on the conflict item, and vice versa. Further studies are needed to determine whether the conflict-item should be rephrased when we aim to measure behavioral addictions that have some kind of social desirability (e.g. exercising, dieting, working), compared to less accepted behavioral addictions (e.g. gaming, eating). The Bergen Workaholic Scale (Andreassen, Griffiths, Hetland, & Pallesen, 2012) has an item addressing negative health consequences as a result of too much work. It might be a better indicator to assess negative consequences of exercise rather than potential interpersonal conflicts.

Our results also showed that fitness exercisers who had an eating disorder were less likely to perceive exercise support from family and friends. This suggests that exercise behavior is less encouraged when it is associated with an eating disorder. Excessive exercise might be the first sign of an eating disorder, and it is an important signal to identify exercise addiction or an eating disorder in its early stages. Approximately 39–48% of people with eating disorders engage in compulsive exercise, indicating a common overlap between the two behaviors (Freimuth, Moniz, & Kim, 2011). Grandi, Clementi, Guidi, Benassi, and Tossani (2011) found that addicted exercisers, especially females, more often than controls presented with dysfunctional eating patterns. Associations between the drive for thinness/muscularity, compulsive exercise, and body dissatisfaction have also been found in a study exploring female fitspiration bloggers (Holland & Tiggemann, 2016). The findings indicate that exercise addiction and eating disorders share some characteristics and may be primary or secondary to each other. If exercise addiction comes first, screening tools need to be reliable and sensitive to the specific features of the phenomenon. It may be difficult to recognize the thin line between passion for exercise versus early signs of exercise addiction if family and friends support excessive exercise patterns.

### 4.1 Limitations

The results of this study should be considered as preliminary in view of some methodological limitations. The data were self-reported, and the study was biased by a lack of validated tools to measure “perceived exercise support from those around me” and “eating disorder present”. The minor change in the EAI-conflict item (including friends and colleagues) should be mentioned as a potential psychometric limitation, but is still in accordance with the theoretical model of behavioral addictions.

The statistical analyses are simple and can only show an indication of differences/similarities between groups. Further exploration is needed to understand the complexity between exercise addiction and social desirability. The results were based on a convenience sample from a cross-sectional survey study, and thus data might have been influenced by common method bias.

In future studies it could also be of interest to investigate whether social support is dependent on other factors such as age, gender, marital status, children, nationality, type of sport (e.g. running), and use of supplements (e.g. protein powder) and anabolic steroids.

## 5 Conclusions

Exercise addiction may be a socially accepted behavior, as fitness exercisers with a high risk of exercise addiction reported the same level of exercise support from relatives as exercisers with low risk. Fitness exercisers reporting high levels of support had lower scores on the EAI conflict-item.

Thus, when assessing exercise addiction, a lack of conflict with family and friends might affect the reliability of the EAI because of the increased risk of false negatives.

Social acceptance of exercise patterns can make it difficult to acknowledge unhealthy exercise, as it is perceived as socially desirable. However, this study also indicated that exercisers with a self-reported eating disorder experienced lower levels of exercise support from their social network, indicating that eating pathology is socially undesirable, whereas exercise pathology in itself is not.
